# Regorafenib plus FOLFIRINOX as first-line treatment for patients with RAS-mutant metastatic colorectal cancer (FOLFIRINOX-R trial): a dose-escalation study

**DOI:** 10.1007/s00280-024-04682-4

**Published:** 2024-07-10

**Authors:** Antoine Adenis, François Ghiringhelli, Ludovic Gauthier, Thibault Mazard, Ludovic Evesque, Alexandre Evrard, Patrick Chalbos, Aurore Moussion, Sophie Gourgou, Marc Ychou

**Affiliations:** 1 Medical Oncology Department, Montpellier Cancer Institute (ICM), INSERM U1194, Montpellier University, Montpellier, 34298 France; 2 Medical Oncology Department, Centre Georges-Francois Leclerc, Dijon, France; 3 Biometrics Unit, Montpellier Cancer Institute (ICM), Montpellier University, Montpellier, France; 4 Medical Oncology Department, Centre Antoine Lacassagne, Nice, France; 5 Montpellier Cancer Research Institute (IRCM), INSERM U1194, Montpellier University, Montpellier, France; 6 Laboratory of Biochemistry and Molecular Biology, Nimes University Hospital, IRCM, University of Montpellier, INSERM, Montpellier, France; 7 Clinical Research and Innovation Department, Montpellier Cancer Institute (ICM), Montpellier University, Montpellier, France

**Keywords:** Regorafenib, FOLFIRINOX, Colorectal cancer, Phase 1 trial, Recommended phase 2 dose

## Abstract

**Purpose:**

The combination of bevacizumab and FOLFIRINOX is used in patients with RAS-mutant metastatic colorectal cancer (RASm-mCRC). Regorafenib, an oral multi-tyrosine kinase inhibitor, has antiangiogenic properties, cytostatic effects and also true cytotoxic effects, unlike bevacizumab. The aim of this study was to determine the maximum tolerated dose (MTD) and the recommended phase 2 dose (RP2D) of the regorafenib-FOLFIRINOX combination in patients with RASm-mCRC.

**Methods:**

The FOLFIRINOX-R trial was a phase 1/2 study where the dose-escalation part (3 + 3 design with three dose levels, DLs) was completed before its early termination. FOLFIRINOX (14-day cycle) included oxaliplatin (standard dose), folinic acid, fluorouracil and irinotecan (150 or 180 mg/m²). Regorafenib (120 or 160 mg daily) was given from day 4 to day 10 of each cycle. Dose-limiting toxicity (DLT) was studied in the first three cycles. Eligibility criteria included ECOG performance status ≤ 1 and not previously treated RASm-mCRC.

**Results:**

Thirteen patients (median age: 65 years; min-max: 40–76) were enrolled. DLT could not be evaluated in one patient (DL3) due to poor observance. The median treatment duration and median follow-up were 6.2 (min-max: 2.3–10) and 13.4 (min-max: 3.8–18.0) months, respectively. Dose was modified in 12/13 (92%) patients. One grade 3 hypokalemia occurred at DL2. MTD was not reached at DL3. Grade 3 diarrhea was recorded in 7/13 patients (13 events) equally distributed in all DLs.

**Conclusion:**

The RP2D for this regorafenib-FFX combination could not be determined due to a high prevalence of grade 3 diarrhea related to treatment as advised by our Independent Data Monitoring Committee.

**Trial registration numbers:**

ClinicalTrials.gov: NCT03828799.

**Supplementary Information:**

The online version contains supplementary material available at 10.1007/s00280-024-04682-4.

## Introduction

Colorectal cancer (CRC) is a major cause of morbidity and mortality in western countries. Most patients will develop metastases and only few will be cured. Palliative chemotherapy, combined or not with targeted agents, can be proposed to patients with unresectable metastases. According to the European Society for Medical Oncology guidelines, the standard first-line treatment in these patients includes doublet (fluoropyrimidines plus oxaliplatin or irinotecan) or triplet chemotherapy (fluoropyrimidines, oxaliplatin and irinotecan) alone or in combination with monoclonal antibodies against epidermal growth factor receptor (EGFR; cetuximab or panitumumab) or vascular endothelial growth factor A (VEGF-A; bevacizumab), depending on the tumor RAS/BRAF mutational status [1].

RAS mutations are identified in about 60% of metastatic CRC (mCRC) and are negative predictors of anti-EGFR antibody efficacy [2]. RAS mutational status is also associated with poor prognosis, as reported in several randomized trials in patients with mCRC treated with doublet chemotherapy with or without bevacizumab [2] or with triplet chemotherapy plus bevacizumab [3, 4]. Currently, the standard first-line treatment of patients with RAS-mutant (RASm) mCRC is doublet or triplet chemotherapy plus bevacizumab [1]. A recent meta-analysis indicated that triplet chemotherapy plus bevacizumab significantly improves the outcome of patients with mCRC (including those with RASm tumors), compared with doublet chemotherapy plus bevacizumab, with only a moderate increase in toxicity [5].

Regorafenib is an oral multi-kinase inhibitor approved for refractory mCRC at the dose of 160 mg per day, 3 weeks on and 1 week off. It inhibits angiogenic and stromal receptor tyrosine kinases, such as VEGFR1/3, PDGFR-b, FGFR-1, and TIE-2, the oncogenic receptor tyrosine kinases c-KIT and RET, and also the activity of signaling kinases, such as RAF1 and B-RAF [6, 7]. Regorafenib displays antiangiogenic properties and also anti-proliferative activities, as demonstrated in colorectal cancer models [6–8] and also in patients with mCRC, with observed clinical responses irrespective of the RAS mutational status or the treatment line [9–13]. In patients with mCRC, regorafenib has been assessed in combination with doublet chemotherapy (FOLFOX and/or FOLFIRI) in two uncontrolled studies [14, 15] and with FOLFIRI in one randomized phase 2 trial [16]. Using a 1 week-on/1 week-off schedule of regorafenib (160 mg orally once per day on days 4–10) with a standard 14-day cycle of FOLFIRI or FOLFOX, Schultheis et al. [14] reported 71% of severe drug-related adverse events (among which 50% were due to neutropenia), including 7% of severe diarrhea, in 45 patients. They concluded that regorafenib has acceptable tolerability in combination with chemotherapy and noticed an increased exposure of irinotecan, but no significant effect on 5-fluorouracil or oxaliplatin pharmacokinetics. Argilés et al. [15] assessed the FOLFOX-regorafenib combination as first-line treatment in a phase 2 trial with 53 patients and reported that the most prevalent severe grade 3–4 treatment-related adverse events were neutropenia (40% including 15% of grade 4), hypertension (28% including 4% of grade 4), peripheral neuropathy (13%), hypophosphatemia (13%) and diarrhea (25%). In this study, 94% of the 53 patients required regorafenib dose modifications (reduction or interruption) due to adverse events. Conversely, the response rate was not increased compared with historical data on patients treated with FOLFOX [15]. Sanoff et al. [16] carried out a randomized phase 2 study to determine whether the addition of regorafenib (160 mg once daily on days 4–10) to FOLFIRI improved progression-free survival (PFS) (versus the placebo-FOLFIRI arm) in 181 patients with mCRC refractory to first-line treatment with oxaliplatin and fluoropyrimidine-based regimens. PFS was longer in the regorafenib-FOLFIRI arm than in the placebo-FOLFIRI arm (median, 6.1 vs. 5.3 months; hazard ratio, 0.73; 95% confidence interval [CI], 0.53–1.01; *p* = 0.056) [16]. Similarly, the overall response rate (ORR) was numerically higher with regorafenib-FOLFIRI (34%; 95% CI, 25-44%) than with placebo-FOLFIRI (21%; 95% CI, 11-33%; *P* = 0.07). However, dose reductions were more frequent in the regorafenib-FOLFIRI than in the placebo-FOLFIRI arm (58% versus 25%). More patients experienced severe adverse events in the regorafenib-FOLFIRI than in the placebo-FOLFIRI arm (79% versus 59%), particularly neutropenia, febrile neutropenia, hypophosphatemia, hand-foot syndrome and diarrhea (15% in the experimental arm). The authors concluded that the regorafenib-FOLFIRI combination was unlikely to provide clinically meaningful improvement for patients with mCRC because of its modest clinical benefit and its toxicity profile [16].

Given the improved outcome observed with triplet chemotherapy plus bevacizumab as first-line treatment of patients with RASm mCRC [5], the need of developing more efficient strategies in this setting, and the hypothesis that regorafenib could do better than bevacizumab when combined with triplet chemotherapy (if adverse events can be controlled), we designed the FOLFIRINOX-R study to assess the safety and the efficacy of regorafenib in combination with FOLFIRINOX [17] in patients with RASm mCRC [18].

## Materials and methods

FOLFIRINOX-R is a prospective, multicentric, non-randomized, open-label, phase 1b/2 trial conducted at four cancer centers in France. It had a standard 3 + 3 design for dose escalation followed by a phase 2 trial to study the safety and efficacy of regorafenib in combination with FOLFIRINOX after identification of the recommended phase 2 dose (RP2D). The study details have been previously reported [18].

### Participants

Key inclusion criteria were: ≥18-year-old patients with an Eastern Cooperative Oncology Group (ECOG) performance status of ≤ 1, with measurable (according to RECIST version 1.1) and unresectable mCRC and RASm by circulating tumor DNA analysis. In addition, patients should not have been previously treated for mCRC. Patients needed to have adequate bone marrow, renal and liver functions, serum lipase ≤ 1.5 times the upper limit of normal (ULN), serum uracil < 17 ng/ml, and wild type homozygous or heterozygous status for the uridine diphosphate glucuronosyltransferase (UGT) 1A1*28 polymorphism [18]. Patients with the UGT1A1*28/*28 genotype were not selected in this FOLFIRINOX-R study.

The study was conducted following the principles of the Declaration of Helsinki and the International Conference on Harmonization and Good Clinical Practice guidelines. The study protocol (ClinicalTrials.gov identifier: NCT03828799), including all amendments, was reviewed and approved by the French ethics committee (CPP Est III) on December 4, 2018, and the French National Agency for the Safety of Health Products (ANSM) on November 21, 2018. Patients provided their written informed consent before enrollment in the study.

### Study design

FOLFIRINOX was administered as per standard: oxaliplatin 85 mg/m² on day 1 (intravenous, IV, infusion over 2 h), immediately followed by folinic acid 400 mg/m² or calcium levofolinate 200 mg/m² (2-hour IV infusion), with the addition of irinotecan (150 or 180 mg/m² ; 90-minute IV infusion through a Y-connector) immediately followed by 5-fluorouracil (400 mg/m² IV bolus then 2400 mg/m² over 46 h continuous infusion) (19). Primary prophylactic granulocyte colony-stimulating factor (G-CSF) was delivered from day 7 to day 12. Regorafenib was administered orally (80, 120, or 160 mg) once per day from day 4 to day 10 of each 14-day cycle. The pre-defined dose levels (DLs) were as follows: DL -1 (irinotecan 150 mg/m² plus regorafenib 80 mg/m²); DL1 (irinotecan 150 mg/m² plus regorafenib 120 mg/m²); DL2 (irinotecan 180 mg/m² plus regorafenib 120 mg/m²); and DL3 (irinotecan 180 mg/m² plus regorafenib 160 mg/m²). Treatment cycles were repeated every 14 days for up to 12 cycles. Then, treatment continued with a maintenance phase (regorafenib alone) until disease progression, unacceptable toxicity, or consent withdrawal. Details on permitted dose delays and modifications were provided in the already published study protocol [18]. Specifically, treatment could be interrupted to perform surgical resection or any regional procedure, if needed. Laboratory and clinical evaluations were performed at each cycle (see reference 18 for more details). During the first three cycles, laboratory assessments were performed also on day 8 of each cycle. Tumor assessment (RECIST, v1.1) was performed every 8 weeks. The main objective of the phase 1 of this study was to determine the maximum tolerated dose (MTD) and the RP2D of the combination of regorafenib plus FOLFIRINOX by testing different doses of regorafenib and irinotecan. The dose-limiting toxicity (DLT) period was defined as the time from the first cycle of treatment until the day of the planned fourth cycle, corresponding to 42 days. DLT was defined as the occurrence of one or more of the following toxicities during the first three cycles of treatment: any unplanned interruption > 7 days of regorafenib due to drug-related toxicity, grade ≥ 3 (CTCAE v5) non-hematologic toxicity (except grade 3 nausea, grade 3 vomiting, grade 3 diarrhea and grade ≥ 3 lipase elevation without signs of pancreatitis), grade ≥ 2 posterior reversible encephalopathy syndrome, grade ≥ 2 retinopathy, any of the following liver-specific toxicities [grade ≥ 3 bilirubin increase, grade ≥ 3 aspartate transaminase (AST) and/or alanine transaminase (ALT) increase, or AST and/or ALT increase > 3 x ULN with concurrent bilirubin increase > 2 x ULN], grade 4 neutropenia lasting > 3 days, grade ≥ 3 febrile neutropenia, grade 4 anemia, platelets < 25,000 /mm^3^ or platelets < 50,000 /mm^3^ with bleeding, grade ≥ 3 international normalized ratio or partial thromboplastin time elevation with bleeding, grade ≥ 3 hemorrhage/bleeding. The phase 2 part of the study would start after the RP2D determination and after the agreement by the Independent Data Monitoring Committee (IDMC). The primary objective of the phase 2 study was to evaluate the 48-week disease-control rate in all treated patients. The main secondary endpoints of the study included safety according to the NCI-CTCAE v.5, ORR according to the RECIST v1.1, deepness of response, disease control rate (DCR), PFS, and overall survival [18].

### Statistical design

A minimum of 12 and a maximum of 24 patients needed to be included in the phase 1 study, with a minimum of 3 and a maximum of 6 patients per DL. Six patients needed to be included at the RP2D. The phase 2 part of the trial followed a Fleming’s one-stage design. Detailed assumptions and numbers of patients to be included were described in Adenis et al. [18].

## Results

### Patient characteristics

Between July 17, 2019 and October 15, 2021, 13 patients with RASm mCRC were enrolled in the phase 1b study. The patient demographics and baseline disease characteristics are summarized in Table 1. Most patients had a left-sided primary tumor (10/13 patients) and were chemotherapy-naive (12/13 patients) (Table 1).

**Table 1:** Patients’ characteristics (phase 1b study)

	Dose level 1	Dose level 2	Dose level 3	ITT sample
ITT population, *n*	3	6	4	13
Safety population^#^, *n*	3	6	4	13
Patients evaluable for DLT^##^, *n*	3	6	3	12
Age, years, median (min; max)	53 (49; 65)	69 (40;76)	64.5 (49;76)	65 (40; 76)
Male/female ratio, *n*	1/2	3/3	2/2	7/6
ECOG PS = 0/1, *n*	1/2	2/4	3/1	6/7
Primary location				
Left side/right side	2/1	4/2	4/0	10/3
Metastatic sites				
Brain Liver Lymph nodes Peritoneum Lung	0 3 0 1 1	1 5 2 1 3	0 4 0 0 1	1 12 3 2 5
Previous chemotherapy	0	1	0	1

ITT: Intention-to-treat, DLT: Dose-limiting toxicity

# Patients who received at least one dose of treatment

## Patients treated and evaluable for DLT (1 unevaluable patient due to low observance)

### Study drug exposure

**Fig. 1:**
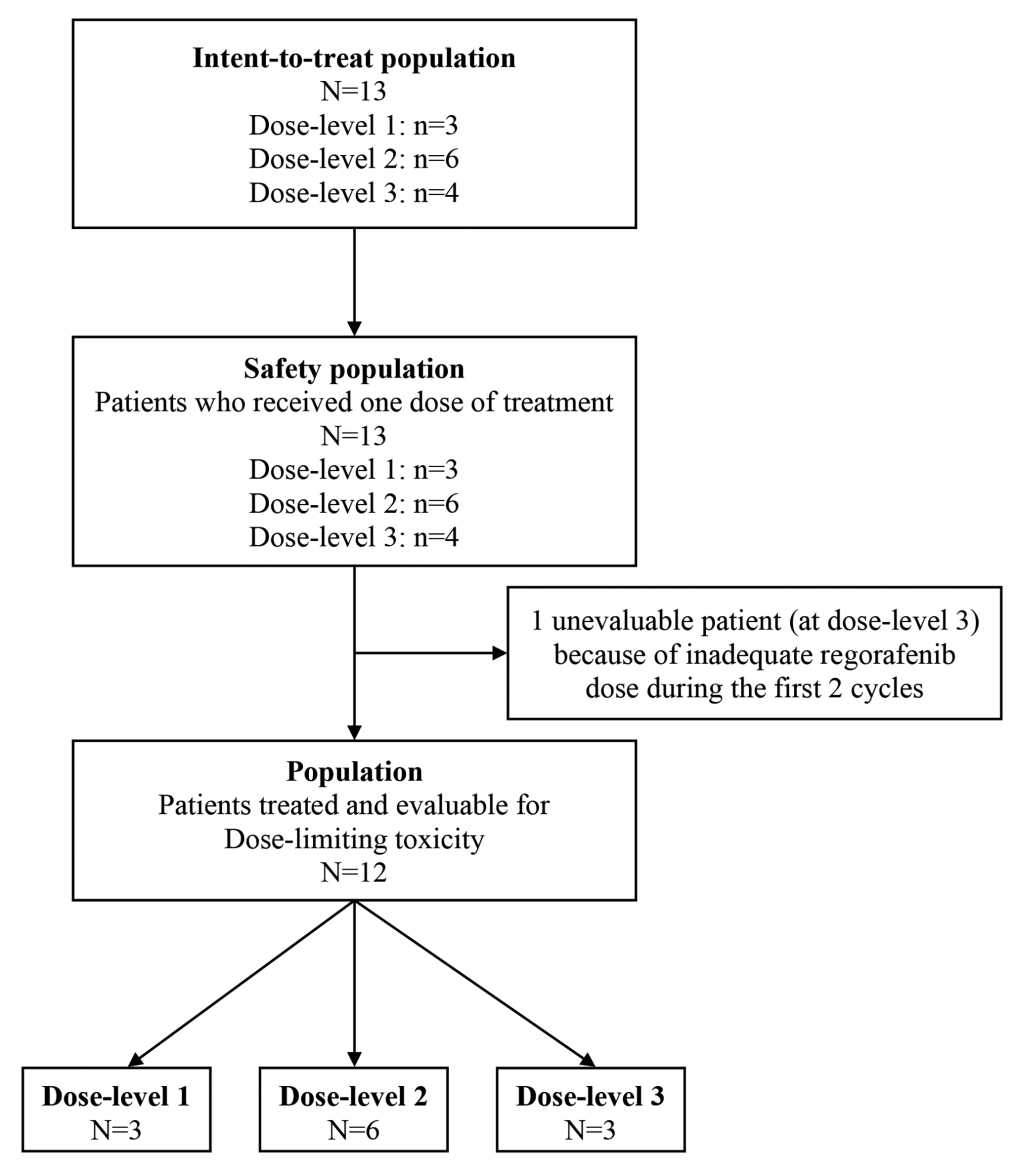
Study flowchart

All patients received at least one dose of the study drug combination (regorafenib plus FOLFIRINOX at one of the three DLs) (Fig. 1). The median number of administered cycles was 12 [interquartile Q1 and Q3: 7.0–15.0] and the median treatment duration was 6.2 months [Q1-Q3: 4.3–7.1]. Additional details on the number of cycles administered and the relative dose intensity per drug and DL are in Table 2.

**Table 2:** Drug exposure

Parameters	Dose level 1 *n* = 3	Dose level 2 *n* = 6	Dose level 3 *n* = 4	Safety sample *n* = 13
**Regorafenib + FOLFIRINOX**				
Treatment duration, months ^a^	4.3 (3.7–6.5)	5.9 (3.5–7.5)	6.6 (5.4-8.0)	6.2 (4.3–7.1)
Number of cycles administered ^a^	7.0 (7.0–12.0)	12.0 (7.0–16.0)	13.5 (11.0-16.5)	12.0 (7.0–15.0)
At least one dose delay ^b^	3 (100)	5 (83.3)	4 (100)	12 (92.3)
At least one dose modification ^b^	3 (100)	5 (83.3)	4 (100)	12 (92.3)
**FU bolus**				
RDI ^a^	0.8 (0.7–0.9)	0.9 (0.7-1.0)	0.9 (0.8-1.0)	0.9 (0.7-1.0)
RDI ≥ 90% ^b^	0	3 (50)	2 (50)	5 (38.5)
At least one dose delay ^b^	3 (100)	3 (50)	2 (50)	8 (61.5)
At least one dose modification ^b^	2 (66.7)	3 (50)	1 (25)	6 (46.2)
**FU continuous infusion**				
RDI ^a^	0.8 (0.6–0.9)	0.9 (0.9-1.0)	0.8 (0.8-1.0)	0.9 (0.6-1.0)
RDI ≥ 90% ^b^	2 (66.7)	6 (100)	3 (75)	11 (84.6)
At least one dose delay ^b^	3 (100)	3 (50)	2 (50)	8 (61.5)
At least one dose modification ^b^	2 (66.7)	2 (33.3)	2 (50)	8 (61.5)
**Oxaliplatin**				
RDI ^a^	0.8 (0.6–0.9)	0.9 (0.7-1.0)	0.8 (0.8–0.9)	0.8 (0.8–0.9)
RDI ≥ 90% ^b^	1 (33.3)	4 (66.7)	3 (75)	8 (61.5)
At least one dose delay ^b^	3 (100)	3 (50)	2 (50)	8 (61.5)
At least one dose modification ^b^	2 (66.7)	4 (66.7)	4 (100)	10 (76.9)
**Irinotecan**				
RDI ^a^	0.8 (0.7–0.9)	0.8 (0.7–0.9)	0.7 (0.7–0.8)	0.8 (0.7–0.8)
RDI ≥ 90% ^b^	2 (66.7)	3 (50)	1 (25)	6 (46.2)
At least one dose delay ^b^	3 (100)	3 (50)	2 (50)	8 (61.5)
At least one dose modification ^b^	3 (100)	4 (66.7)	4 (100)	11 (84.6)
**Regorafenib**				
RDI ^a^	0.9 (0.5–0.9)	0.9 (0.7-1.0)	0. (0.5–0.8)	0.8 (0.6–0.9)
RDI ≥ 90% ^b^	2 (66.7)	4 (66.7)	1 (25)	7 (53.8)
At least one dose delay ^b^	3 (100)	1 (16.7)	1 (25)	5 (38.5)
At least one dose modification ^b^	1 (33)	4 (66.7)	3 (75)	8 (61.5)

^a^ Data reported as median (Q1-Q3)

^b^ Data reported as *n* (%)

RDI: Relative dose intensity; FU: 5-fluorouracil

Eight patients (62%) required at least one dose delay (3 at DL1; 1 at DL2, and 2 at DL3) and twelve (92%) needed a dose reduction (3 at DL1; 5 at DL2, and 4 at DL3). The most common cause of dose modification was toxicity of treatment (58/69 cycles with a dose modification). Four patients received maintenance therapy following the first 12 cycles of treatment.

At the time of data cut-off, all patients but one had terminated the study treatment. Reasons for treatment discontinuation were progressive disease (*n* = 4), toxicity (*n* = 2, treatment interruption > 28 days: 1, several episodes of grade 3 diarrhoea: 1), and intent for a curative local treatment (*n* = 6).

### Safety analyses

Treatment-related adverse events of any grade were reported in all 13 patients (Table 3). Grade ≥ 3 treatment-related adverse events were reported for almost all patients (*n* = 12), particularly diarrhea (seven [53.8%]), neutropenia (two [15.4%]), and peripheral neuropathy (two [15.4%]) (Table 4). Among these grade ≥ 3 treatment-related adverse events, 13 grade 3 diarrhea episodes were reported by 7/13 patients despite standard procedures including patient selection (dihydropyrimidine dehydrogenase phenotyping and UGT1A1 genotyping, therapeutic education, use of high-dose loperamide, oral and/or IV fluids, hospitalization, and per protocol dose modifications). The median duration of these events was 8 days [min-max: 2-121-11] in six evaluable patients. No relationship between the occurrence of grade ≥ 3 diarrhea episodes and UGT1A1*28 genotype (either wild type homozygous or heterozygous status) could be identified (data not shown). There was no treatment-related death. One DLT (e.g. grade 3 hypokalemia) was recorded at DL2 and one grade 4 neutropenia was reported at DL1. The latter was not considered to be another DLT because the prophylactic G-CSF injection had been unintentionally omitted. As per protocol, MTD was not reached and RP2D should have been the DL3. However, our IDMC highlighted the high frequency of grade 3 diarrhea at all DLs. Accordingly, the IDMC could not validate the DL3 as the RP2D and recommended stopping the study and not proceeding with the phase 2 part.

**Table 3:** Treatment-related adverse events

	Dose level 1 (IRI 150 mg/m² REGO 120 mg)		Dose level 2 (IRI 180 mg/m² REGO 120 mg)		Dose level 3 (IRI 180 mg/m² REGO 160 mg)		Total	
	Patients *N* = 3	TRAE *N* = 44	Patients *N* = 6	TRAE *N* = 110	Patients *N* = 4	TRAE *N* = 82	Patients *N* = 13	TRAE *N* = 236
Anemia	0	0	3 (50%)	7	2 (50%)	3	5 (38.5%)	10
Neutropenia	3 (66.7%)	6	1 (16.7%)	1	0	0	4 (30.8%)	7
Thrombopenia	1 (33.3%)	2	1 (16.7%)	2	0	0	2 (15.4%)	4
Coronary vasospasm	0	0	1 (16.7%)	1	0	0	1 (7.7%)	1
Vertigo	0	0	0	0	1 (25%)	1	1 (7.7%)	1
Abdominal pain	1 (33.3%)	1	1 (16.7%)	1	1 (25%)	2	3 (23.1%)	4
Anorectal discomfort	0	0	0	0	1 (25%)	1	1 (7.7%)	1
Stomatitis	0	0	1 (16.7%	1	0	0	1 (7.7%)	1
Constipation	1 (33.3%)	1	1 (16.7%)	1	0	0	2 (15.4%)	2
Diarrhea	2 (66.7%)	7	6 (100%)	23	4 (100%)	21	12 (92.3%)	51
Dyspepsia	0	0	0	0	1 (25%)	1	1 (7.7%)	1
Gingival bleeding	0	0	1 (16.7%)	1	0	0	1 (7.7%)	1
Nausea	0	0	4 (66.7%)	10	4 (100%)	7	8 (61.5%)	17
Salivary hypersecretion	0	0	0	0	1 (25%)	1	1 (7.7%)	1
Subileus	0	0	1 (16.7%)	1	0	0	1 (7.7%)	1
Vomiting	1 (33.3%)	3	1 (16.7%)	1	2 (50%)	4	4 (30.8%)	8
Asthenia	2 (66.7%)	9	6 (100%)	10	3 (75%)	10	11 (84.6%)	29
Mucositis	1 (33.3%)	1	3 (50%)	5	0	0	4 (30.8%)	6
Cholestasis	0	0	1 (16.7%)	1	0	0	1 (7.7%)	1
Anaphylactic reaction	0	0	0	0	1 (25%)	1	1 (7.7%)	1
Drug hypersensitivity	0	0	1 (16.7%)	1	0	0	1 (7.7%)	1
Paronychia	0	0	1 (16.7%)	1	0	0	1 (7.7%)	1
Pharyngitis	0	0	1 (16.7%)	1	0	0	1 (7.7%)	1
Transaminase increase	0	0	1 (16.7%)	1	1 (25%)	1	2 (15.3%)	2
LDH increase	0	0	0	0	1 (25%)	1	1 (7.7%)	2
Glutamyltransferase increase	0	0	0	0	1 (25%)	2	1 (7.7%)	1
Thrombocythemia	0	0	0	0	1 (25%)	2	1 (7.7%)	2
Weight loss	1 (33.3%)	4	3 (50%)	4	3 (75%)	4	7 (53.8%)	12
Decrease appetite	1 (33.3%)	1	3 (50%)	12	3 (75%)	3	7 (53.8%)	16
Hypoalbuminemia	0	0	1 (16.7%)	1	0	0	1 (7.7%)	1
Hypokalemia	0	0	3 (50%)	4	0	0	3 (23.1%)	4
Hypomagnesemia	0	0	1 (16.7%)	1	0	0	1 (7.7%)	1
Arthralgia	1 (33.3%)	1	0	0	0	0	1 (7.7%)	1
Trismus	1 (33.3%)	1	0	0	0	0	1 (7.7%)	1
Dizziness	0	0	1 (16.7%)	1	0	0	1 (7.7%)	1
Dysgeusia	0	0	2	2	0	0	2 (15.4%)	2
Dysphonia	0	0	1 (16.7%)	1	0	0	1 (7.7%)	1
Peripheral neuropathy	1 (33.3%)	3	4 (66.7%)	7	3 (75%)	9	8 (61.5%)	19
PPE	0	0	1 (16.7%)	1	1 (25%)	1	2 (15.4%)	2
Proteinuria	0	0	1 (16.7%)	1	1 (25%)	1	2 (15.4%)	2
Epistaxis	1 (33.3%)	1	0	0	0	0	1 (7.7%)	1
Pulmonary Embolism	0	0	1 (16.7%)	1	0	0	1 (7.7%)	1
Rhinorrhea	0	0	1 (16.7%)	1	0	0	1 (7.7%)	1
Alopecia	2 (66.7%)	2	2 (33.3%)	2	2 (50%)	2	6 (46.2%)	6
Dry skin	1 (33.3%)	1	1 (16.7%)	1	0	0	2 (15.4%)	2
Hypertension	0	0	0	0	2 (50%)	3	2 (15.4%)	3

IRI: irinotecan; REGO: regorafenib; TRAE: Treatment-related adverse events; PPE: Palmar-plantar erythrodysesthesia

**Table 4:** Treatment-related grade 3–4 adverse events

	Dose level 1 (IRI 150 mg/m² REGO 120 mg)		Dose level 2 (IRI 180 mg/m² REGO 120 mg)		Dose level 3 (IRI 180 mg/m² REGO 160 mg)		Total	
	Patients *N* = 3	AE *N* = 5	Patients *N* = 6	AE *N* = 9	Patients *N* = 4	AE *N* = 9	Patients *N* = 13	AE *N* = 23
Diarrhea	1 (33.3%)	3	2 (33.3%)	4	4 (100)	6	7 (53.8%)	13
Neutropenia	1 (33.3%)	1	1 (16.7%)	1	0	0	2 (15.4%)	2
Peripheral neuropathy	0	0	1 (16.7%)	1	1 (25%)	1	2 (15.4%)	2
Anaphylactic reaction	0	0	0	0	1 (25%)	1	1 (7.7%)	1
Loss of appetite	0	0	1 (16.7%)	1	0	0	1 (7.7%)	1
Hypokalemia	0	0	1 (16.7%)	1	0	0	1 (7.7%)	1
Pulmonary embolism	0	0	1 (16.7%)	1	0	0	1 (7.7%)	1
Hypertension	0	0	0	1	1 (25%)	1	1 (7.7%)	1

IRI: irinotecan; REGO: regorafenib; AE: adverse events

### Efficacy

Efficacy was evaluated in all 13 patients. At data cut-off, the median follow-up period was 30 months (range 6–40). In the intention-to-treat population (*n* = 13), a partial response was observed in 8 patients (61.5% [95% CI: 31.6–86%]) and DCR was 100% (Table 5). The median deepness of response was − 52.5% (min-max: -1.1-73%) (Supplementary Table). Six patients underwent surgery because metastases became resectable following treatment and another one underwent thermal ablation (Supplementary Table). The median PFS was 10 months (95% CI: 7.8–15.4).

**Table 5:** Summary of the outcomes

	Dose level 1 (IRI 150 mg/m² REGO 120 mg)	Dose level 2 (IRI 180 mg/m² REGO 120 mg)	Dose level 3 (IRI 180 mg/m² REGO 160 mg)	ITT sample
Response				
Response Complete response Partial response, *n* (%) Stable disease, *n* (%)	0 2 (66.7) 1 (33.3)	0 3 (50) 3 (50)	0 4 (100) 0	0 9 (69.2) 4 (30.8)
Disease control rate, *n* (%) Progressive disease, *n*	3 (100) 0	6 (100) 0	0 0	13 (100) 0
Median PFS (95%CI), months	N/A	N/A	N/A	9.1 (7.5-NE)
Median OS (95%CI), months	N/A	N/A	N/A	18 (9.9-NE)

IRI: irinotecan; REGO: regorafenib; PFS: progression-free survival; OS: overall survival; ITT: intention-to-treat; NE: not evaluable

## Discussion

The aim of the multicenter FOLFIRINOX-R study was to determine the MTD and RP2D of the regorafenib plus FOLFIRINOX combination in patients with RASm mCRC. The study did not meet its primary endpoint. Since the MTD was not reached, the IDMC advised us not to recommend any RP2D due to the frequent occurrence of severe diarrhea (> 50% of patients). The IDMC also recommended to interrupt the study before moving to the phase 2. Severe diarrhea was previously reported in patients treated with any of the drugs included in our experimental regimen: regorafenib (17%) [9], FOLFOX/FOLFIRI (11–14%) [19] and FOLFOXIRI/FOLFIRINOX (20–29%) [17, 20]. Moreover, severe diarrhea in patients with mCRC is not infrequent when chemotherapy is combined with antiangiogenic agents: FOLFOX/FOLFIRI with bevacizumab (8%) [5], FOLFIRI with regorafenib (10–15%) [14, 16], FOLFOX with regorafenib (4–23%) [14, 15], and FOLFOXIRI/FOLFIRINOX with bevacizumab (18–20%) [5, 21]. However, we did not anticipate its occurrence in > 50% of patients receiving the regorafenib-FOLFIRINOX combination. We were fully aware that regorafenib inhibits glucuronosyltransferases, particularly UGT1A1. Moreover, Schultheis et al. [14] showed that when combined with irinotecan, regorafenib increases the exposure of its active metabolite SN-38. Therefore, we excluded patients who carried the UGT1A1*28/*28 variant and who are at high-risk of irinotecan-induced severe diarrhea [22, 23]. Like others [14, 16], we used an intermittent regime of regorafenib (from day 4 to day 10) rather than the standard 3 weeks on and 1 week off regimen in order to avoid the concomitant exposure to regorafenib and irinotecan. Also, the starting doses of regorafenib and irinotecan were lower (120 mg and 150 mg/m², respectively) than previously used in regorafenib-irinotecan combinations (160 mg and 180 mg/m², respectively) [14, 16]. Finally, we carried out dihydropyrimidine dehydrogenase phenotyping (exclusion of patients with plasma uracil concentration ≥ 16 ng/ml) to avoid severe adverse events related to the use of fluorouracil, including diarrhea [24]. Lastly, we preferentially used FOLFIRINOX as triplet chemotherapy rather than FOLFOXIRI (21) because the irinotecan dose is lower in FOLFIRINOX (150 mg/m²) than in FOLFOXIRI (165 mg/m²) and due to our long experience with FOLFIRINOX for colorectal and pancreatic cancer management (metastatic and adjuvant settings) [17, 21, 25, 26]. Therefore, we thought that we took all preventive measures to limit the occurrence of severe diarrhea. Our inability to reach our primary objective could be explained by the fact we had more than expected occurrence of grade 3 diarrhea as a DLT in the study design.

Although we interrupted the study before the phase 2 part, we obtained some efficacy results based on the 13 patients included in the phase 1b. The DCR (100%), ORR (66% ORR) and deepness of response rate (-52.5%) were impressive (especially for patients with RASm mCRC), but apparently without any added value compared with the findings of the TRIBE study on FOLFOXIRI plus bevacizumab in 252 patients among whom 40% had RASm mCRC (DCR: 86.1%, ORR: 65.1%, deepness of response rate: -42.2%) [3]. The median PFS were 10 months in our trial and 12.1 months (95% CI: 10.9–13.2) in the TRIBE study [3], with similar median treatment duration (∼ 6 months) in both trials. Similarly to the conclusions from the trials on the regorafenib-FOLFOX or FOLFIRI combination [14, 15], we are unable from this study to suggest that regorafenib plus FOLFIRINOX will provide a greater benefit compared with other combinations of antiangiogenic antibody plus triplet chemotherapy (e.g. bevacizumab plus FOLFOXIRI) as first-line treatment of mCRC. However, the sample size for this study was small and since focus was on safety, a more definitive conclusion cannot be made at this point.

## Conclusion

FOLFIRINOX-R did not meet its primary objective because we could not determine the RP2D of regorafenib combined with FOLFIRINOX. More than half of patients had one or more grade 3 diarrhea episodes, regardless of the regorafenib and irinotecan dose levels. Despite a 100% disease control rate, we are unable to conclude that this combination would provide superior benefits to standard triplet chemotherapy plus bevacizumab in RASm mCRC.

## Electronic supplementary material

Below is the link to the electronic supplementary material.

Supplementary Table: Deepness of response (DpR) and surgery after treatment initiation

## Data Availability

The corresponding author will provide data and datasets generated and/or analyzed during the study upon reasonable request.
