# Supplementary material for: Regorafenib plus FOLFIRINOX as first-line treatment for patients with RAS-mutant metastatic colorectal cancer (FOLFIRINOX-R trial): a dose-escalation study
Source: Cancer Chemother Pharmacol. 2024 Jul 10;94(3):443–52. doi: 10.1007/s00280-024-04682-4 (PMC11420384; doi:10.1007/s00280-024-04682-4)
Supplement: Supplementary file 1 — Supplementary Table: Deepness of response (DpR) and surgery after treatment initiation [file 280_2024_4682_MOESM1_ESM.docx]

CANCER CHEMOTHERAPY AND PHARMACOLOGY

**Regorafenib plus FOLFIRINOX as first-line treatment for patients with RAS-mutant metastatic colorectal cancer (FOLFIRINOX-R trial): a dose-escalation study**

Antoine Adenis, François Ghiringhelli, Ludovic Gauthier, Thibault Mazard, Ludovic Evesque, Alexandre Evrard, Patrick Chalbos, Aurore Moussion, Sophie Gourgou, Marc Ychou.

Corresponding author: Prof Antoine Adenis, Department of Medical Oncology, Montpellier Cancer Institute (ICM), 34298 Montpellier, France. Email: antoine.adenis@icm.unicancer.fr

**Supplementary Table** Deepness of response and surgery after treatment initiation

| **Center/Patient identifier** | **Dose level** | **DpR (%)** | **Subsequent surgery** |
| --- | --- | --- | --- |
| 1-001 | 1 | -55.3 | Colectomy, salpingo-oophorectomy, omentectomy |
| 1-002 | 1 | -21.7 | Proctectomy, liver surgery |
| 1-003 | 1 | -64.7 | Liver surgery |
| 1-004 | 2 | -1.1 | No |
| 2-001 | 2 | -67.7 | No |
| 2-002 | 2 | -32.4 | No |
| 2-003 | 2 | -49.3 | No |
| 2-004 | 2 | -22.3 | No |
| 3-001 | 2 | -52.5 | No |
| 1-005 | 3 | -59.2 | Lung thermal ablation |
| 1-006 | 3 | -40.6 | Proctectomy |
| 2-005 | 3 | -73 | Liver surgery |
| 2-006 | 3 | -62.3 | Liver surgery |

DpR: Deepness of response
